# Supplementary material for: Unlocking Overexpressed Membrane Proteins to Guide Breast Cancer Precision Medicine
Source: Cancers (Basel). 2024 Apr 3;16(7):1402. doi: 10.3390/cancers16071402 (PMC11011122; doi:10.3390/cancers16071402)
Supplement: Supplementary file 1 [file cancers-16-01402-s001.zip › cancers-2841087-supplementary.pdf]

Supplementary Table S1. List of antibodies used and their respective dilutions.

Antibody	Company	Catalog	Dilution
ANTI-LRRC15	Sigma-Aldrich	HPA035503	1:400
ANTI-EFNA3	Sigma-Aldrich	SAB1401123	1:400
ANTI-TSPAN13	Abcam	ab121262	1:200
ANTI-CA12	Abcam	ab218983	1:200

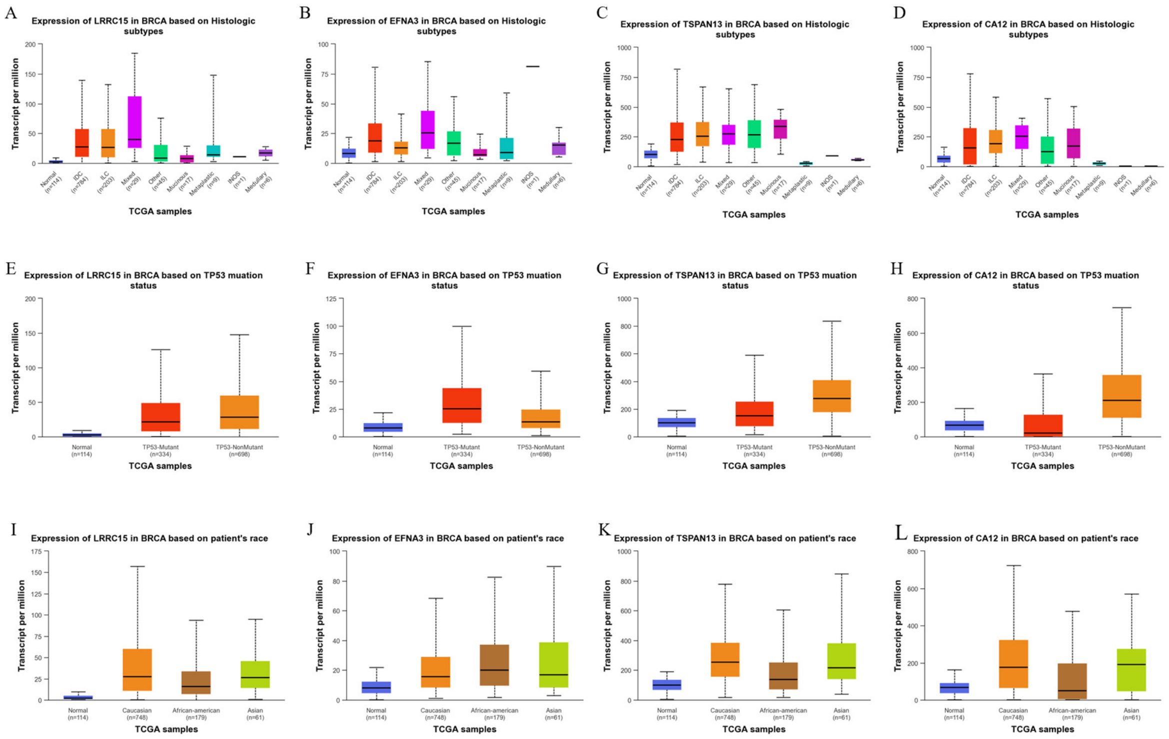

Supplementary Figure S1. Gene expression of the four target genes in relation to the histologic subtype, TP53 mutation and race patients. The y-axis represents the level of transcript per millon and the x-axis represents the samples based on patient`s race, histologic

subtypes and TP53 mutation status. (A,E,I) LRRC15, (B,F,J) EFNA3, (C,G,K) TSPAN13, (D,H,L) CA12.

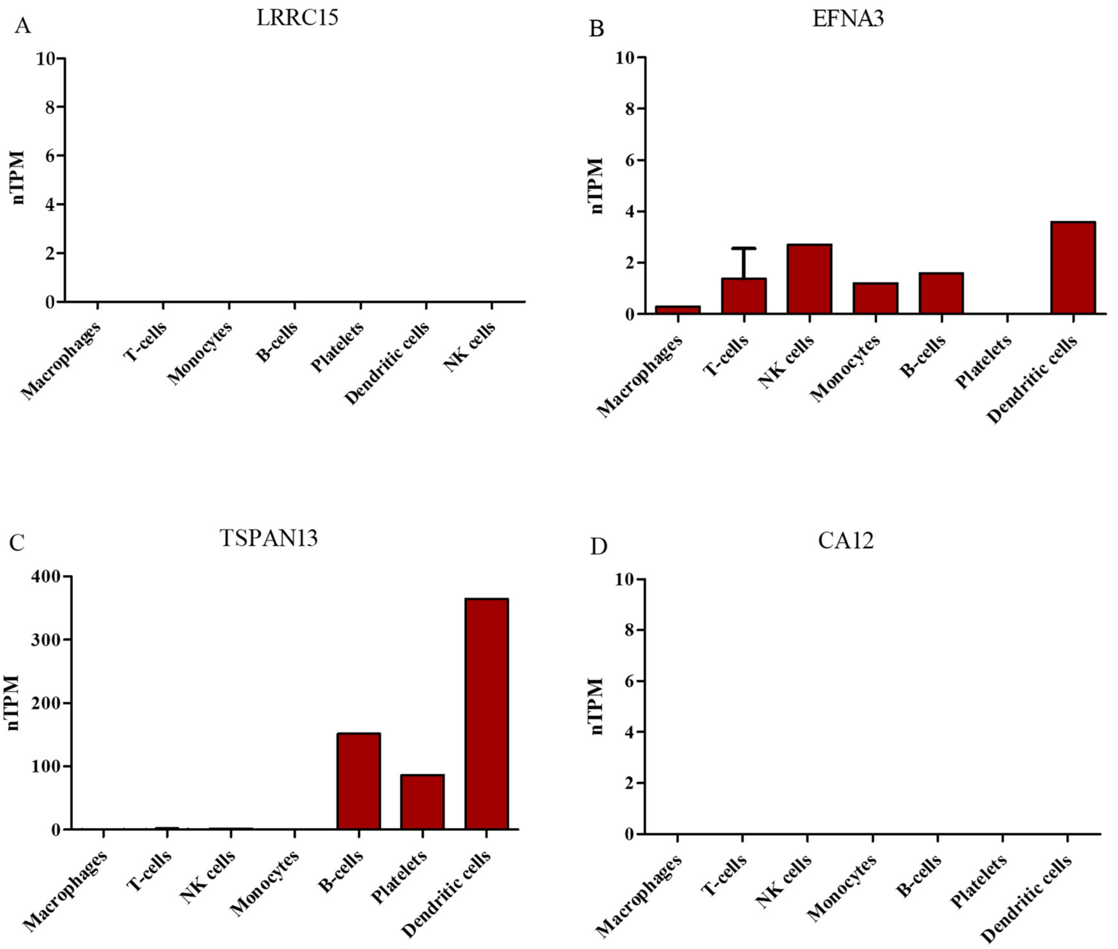

Supplementary Figure S2. Single cell RNA (scRNA) gene expression of the 4 targets in PBMC cells. The y-axis represents the normalized expression (nTPM) of the scRNA and the x-axis represents the different samples included in the study. (A) LRRC15, (B) EFNA3, (C) TSPAN13, (D) CA12.

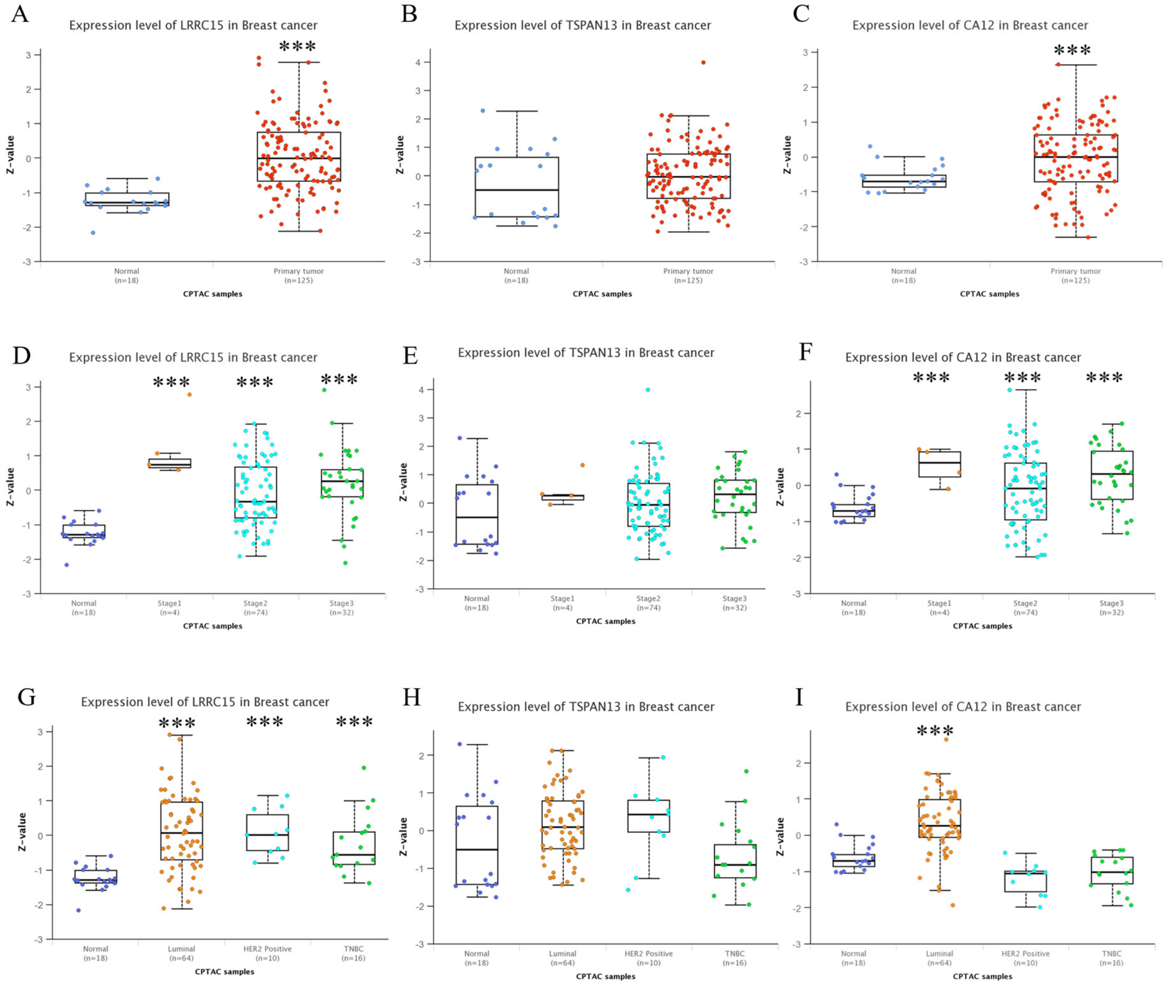

Supplementary Figure S3. Protein expression profiles of LRRC15, TSPAN13, and CA12 targets in BC from UALCAN database. Protein expression profiles of LRRC15, TSPAN13, and CA12 targets in the context of breast cancer. The comparison is as follows: (A–C) Normal samples (n=18) vs. Primary tumor samples (n=125). Blue bar: Normal samples. Red bar: Primary tumor samples. (D–F) Normal samples vs. Tumor staging. Orange bar: Stage 1 samples (n=4). Blue bar: Stage 2 samples (n=74). Green bar: Stage 3 samples (n=32). (G–I) Normal samples vs. Different molecular subtypes of breast cancer. Orange bar: Luminal samples (n=64). Blue bar: HER2 positive samples (n=10). Green bar: Triple Negative samples (n=16). Statistical significance was indicated by $p<0.01$ (***) levels. Non-tumor breast tissue (Normal) was utilized as the statistical reference.

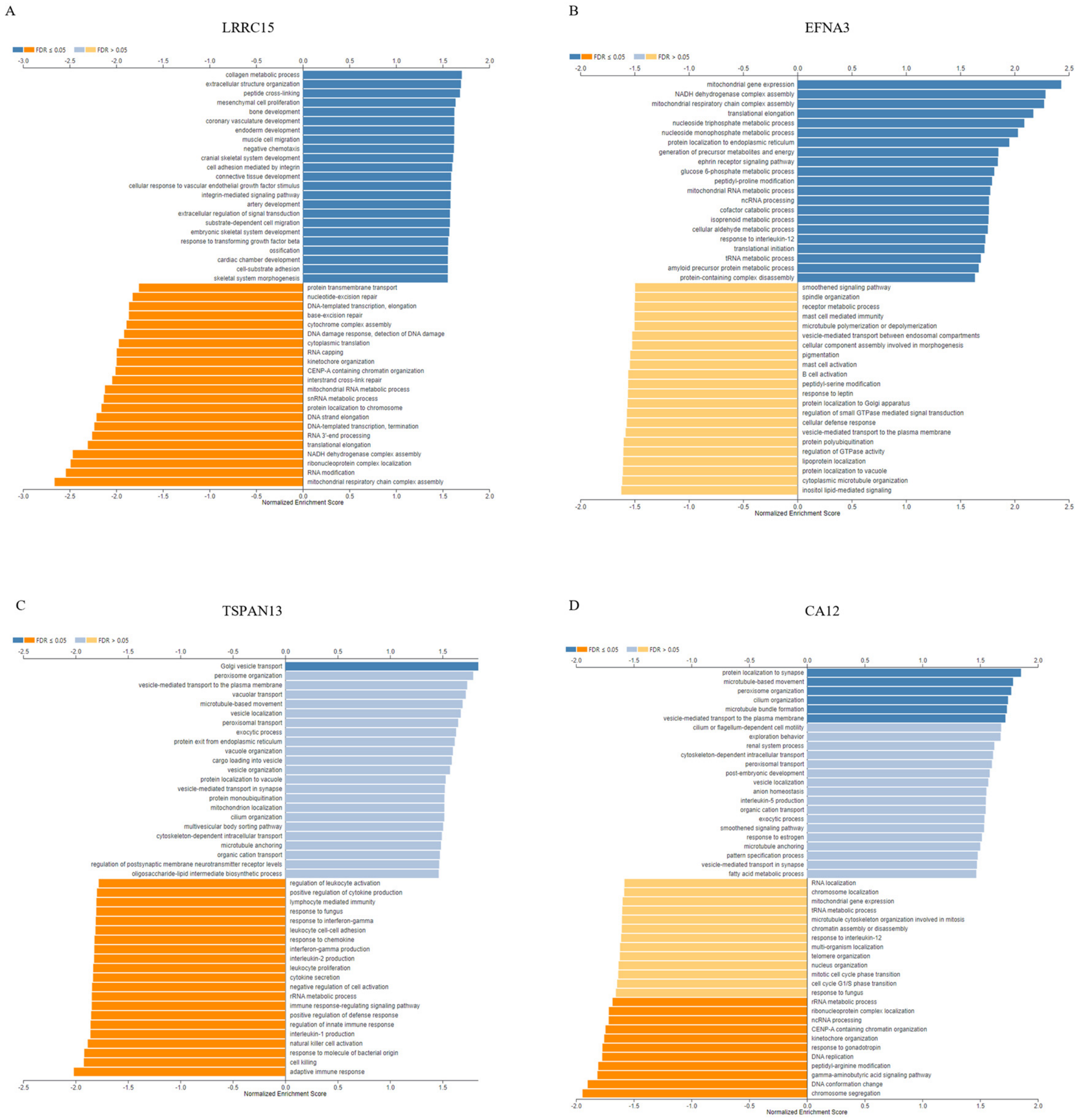

Supplementary Figure S4: Enrichment plots from gene set enrichment analysis (GSEA). Visualization of (A) LRRC15, (B) EFNA3, (C) TSPAN13, (D) CA12 enriched pathways through LinkedOmics (https://linkedomics.org) in TCGA breast cancer platform.
